# Sensory Profiles and Consumer Acceptance of New Culinary Sorghum Varieties in Cooked Form

**DOI:** 10.3390/foods15213915

**Published:** 2026-11-01

**Authors:** Sujita Lama, Melinda Yerka, Yinping Jiao, Xiaofen Du

**Affiliations:** 1 Department of Nutrition and Food Sciences, Texas Woman’s University, 304 Administration Dr, Denton, TX 76209, USA; slama2@twu.edu; 2 Department of Agriculture, Veterinary, & Rangeland Sciences, University of Nevada-Reno, 1664 N. Virginia St., Reno, NV 89557, USA; myerka@unr.edu; 3 Institute of Genomics for Crop Abiotic Stress Tolerance (IGCAST), Department of Plant and Soil Science, Texas Tech University, 2500 Broadway, Lubbock, TX 79409, USA; yijiao@ttu.edu

**Keywords:** *Sorghum bicolor* (L.) Moench, sorghum sensory profile, consumer acceptance, hedonic evaluation, sorghum breeding

## Abstract

Despite growing interest in sorghum as a nutritious and sustainable grain, the use of sorghum grain for direct human consumption in the United States remains limited, partly due to its less desirable sensory characteristics compared to more common cereal crops. A new breeding program was initiated in Nevada in 2017 to focus on improving the culinary qualities of sorghum. This study aimed to investigate consumer (*n* = 220) responses using 6 hedonic ratings and 19 sensory attribute intensities for nine cooked sorghum grain samples (seven new breeding lines and two hybrid controls). The results showed that the majority of samples received positive hedonic scores (>5.0, 9-point hedonic scale) for “overall liking” and the liking of overall aroma, taste, texture, aftertaste, and mouthfeel. The predominant sensory attributes (intensities > 4.0, 0–10 line scale) included four aromas (starchy, earthy, nutty, fresh), one taste (bland), and three textures (residual particles, hardness, roughness). Pearson’s correlation analysis showed that consumer overall liking was positively associated with the liking of overall aroma, taste, texture, aftertaste, and mouthfeel, as well as perceived intensity of fresh aroma and sweet taste. In contrast, overall liking was negatively associated with the intensities of earth, grassy, and musty aromas; bitter and astringent flavor; and hardness, residual particles, and roughness texture. Among the nine samples, LUB24102, LUB24112, and LUB24107 were the three most preferred, whereas UC24007 received the lowest overall liking score. This study is the first to incorporate consumer input to evaluate these newly developed sorghum varieties in cooked form. The findings provide a foundation for ongoing culinary sorghum breeding to develop new cultivars and hybrids with sensory characteristics that better meet consumer preferences and expectations of cooked sorghum grain.

## 1. Introduction

Globally, sorghum (*Sorghum bicolor* L. Moench) is the fifth most widely cultivated cereal crop. It is prized for its versatility as a cereal grain and bioenergy crop and its ability to thrive in harsh environments [1,2,3]. Its resilience to drought, ability to grow in nutrient-poor soils, and minimal need for fertilization make it a vital staple crop in regions facing food security challenges, mainly in drought-prone areas of the world. As declining water availability continues to threaten agriculture, sorghum’s adaptability has drawn attention for its potential to ensure food security [4,5,6]. Based upon usage and production characteristics, sorghum is divided into four major categories: biomass sorghum (for textile and paper production), sweet sorghum (for syrup and sugar from stalks), forage sorghum (or fodder), and grain sorghum (food, feed, and ethanol) [7,8,9].

Grain sorghum is the main type used for ethanol production, animal feeds, and human food. It is consumed as whole cooked grain or processed into flour, porridge, breads, snacks, and other foods [10,11,12]. Sorghum grain may be processed into semolina (rawa or suji), flaked, popped, or puffed to increase its versatility for food preparation [10,13,14]. Sorghum flour is widely used in gluten-free tortillas, breads, cakes, pancakes, biscuits, cookies, pasta, and noodles, providing an alternative to wheat-based products. Innovations such as sourdough fermentation and protein fortification can further improve the sensory and nutritional properties of sorghum-based baked products [10,15]. Sorghum has also gained increasing attention in gluten-free pasta and noodle applications because of its nutritional value and phenolic content. Because sorghum requires less water to grow than many other crops, sorghum-based bakery products may offer additional life cycle energy and water advantages, including cost-effectiveness, extended shelf life, and broad potential for product development [10,16,17]. In addition, sorghum grain can be cooked directly by boiling, steaming, or pressure cooking and consumed as a whole grain or incorporated into porridges and other dishes [11,12,18].

Despite the diverse food applications of sorghum, much of the existing research has focused on processed sorghum products, particularly flour-based, baked, extruded, popped, and fermented foods. Comparatively less attention has been given to whole sorghum grain intended for direct consumption after cooking. In particular, limited information is available on the sensory characteristics and flavor compounds of cooked sorghum grains and how these characteristics influence consumer acceptance [4,16,18,19]. A better understanding of the sensory attributes that drive consumer liking or disliking without the addition of other functional ingredients that could confound human sensory ratings such as for flavor and texture is therefore important for guiding sorghum breeding and developing grain varieties with improved eating quality, including for direct human consumption.

Sorghum grain is nutrient dense, containing carbohydrates (50–80%), protein (8–18%), lipids (1.6–7.6%), dietary fiber (~19%), ash (1.3–3.3%), vitamins (B complex), and minerals (potassium and phosphorus) [7,15,20,21]. Sorghum proteins include albumins, globulins, glutelins, and kafirins, but lack the gluten-forming proteins gliadin and glutenin, making sorghum naturally gluten-free. Its carbohydrates are predominantly starch, consisting of approximately 70–80% amylopectin and 20–30% amylose [7,10,22]. while it also contains relatively high levels of resistant starch compared with maize and wheat. These characteristics, together with growing interest in non-wheat grains, sustainable food systems, and functional foods, have increased the demand for sorghum-based products [23,24,25,26].

Sorghum breeding has traditionally emphasized agronomic traits such as drought tolerance, heat resistance, high yield, and pest resilience due to the crop’s importance in semi-arid regions [5,27,28]. More recently, breeding efforts have expanded to include nutritional quality, food processing performance, and consumer-preferred sensory characteristics [29,30]. Traits such as endosperm chemistry and structure, grain hardness, tannin and phenolic content, sucrose synthesis profiles, and flavor-active compounds have received increasing attention because of their potential effects on processing quality, taste, aroma, and post-cooking texture [31,32,33]. Low-tannin varieties are often preferred for their milder flavor, whereas pigmented sorghums with higher phenolic contents are increasingly targeted for functional foods because of their antioxidant properties [10,16,34]. Despite these advances, a significant research gap remains in linking sorghum breeding traits with sensory characteristics and consumer acceptance, particularly for directly cooked whole grains.

This study provides comprehensive information on the sensory characteristics and consumer acceptance of the newly developed sorghum varieties based on consumer testing. The varieties (seven cultivars and two hybrids) are some of the first germplasms released from the new culinary sorghum breeding program at the University of Nevada, Reno, which was established in 2017. Phenotypic differences during the breeding process were selected to create wide variation in grain composition and physicochemical properties, including starch and protein composition, phenolic and tannin content, sugar profiles, grain hardness, and endosperm characteristics, which ultimately influence eating quality. Cooking further modifies these inherent characteristics through starch gelatinization, protein denaturation, Maillard reactions, and the release, loss, or formation of volatile and non-volatile flavor compounds. Genetic and cooking-related factors collectively determine the aroma, taste, texture, mouthfeel, and overall acceptability of cooked sorghum grain. Therefore, characterizing consumer responses to newly developed culinary sorghum varieties is important for identifying desirable and undesirable sensory attributes and providing practical information to guide ongoing selection of sorghum varieties in breeding programs with improved eating quality and greater potential for direct human consumption.

## 2. Materials and Methods

### 2.1. Sorghum Grain Samples and Cooking

A total of nine sorghum samples were used for this study, including two hybrids and seven cultivars. The seven cultivars included three grown on a private farm in Lubbock, TX in 2024 (LUB24112, LUB24102, LUB24017) and four grown at the University of California-Davis Hutchison research farm in 2024 (UC24004, UC24007, UC24109, and UC24001). The two hybrid cultivars included UC24059, which is ‘G6,’ a commercial bronze-seeded control hybrid from Richardson Seeds (Vega, TX, USA), and UC24011, which is ‘A.N641 × R.N642,’ a publicly available, bronze-seeded, waxy (low-amylose) hybrid released in 2015 that exhibits no yield penalty with the waxy trait. The hybrid cultivars are established commercial or adapted sorghum hybrids included as reference standards for comparison with the experimental breeding lines. All grain samples were provided by the University of Nevada, Reno sorghum breeding program. All sorghum was produced using conventional agronomic methods. Varieties were planted in May (LUB24) or June (UC24), grown in the summer, and harvested in October–November, 2024). About 5.0 lbs (~2.3 kg) of threshed and cleaned sorghum grain samples for each variety were shipped to the senior author’s lab for consumer testing.

**Figure 1:**
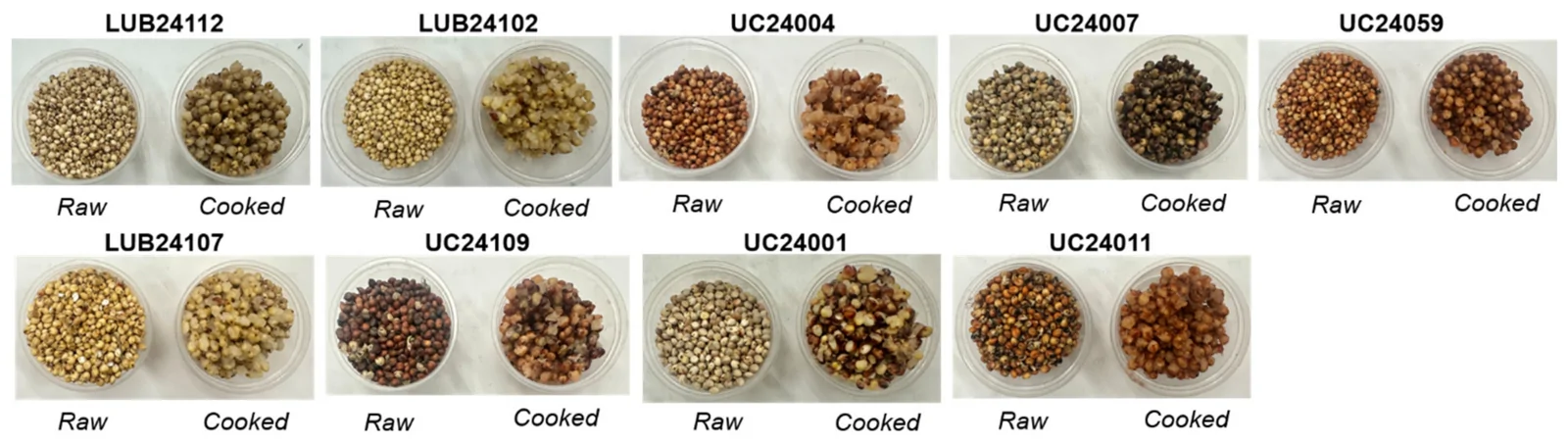
Nine sorghum grain samples in both raw and cooked forms.

Before finalizing the cooking method and conducting the sensory study, several preliminary cooking trials were carried out to evaluate the effects of grain soaking time and grain-to-water ratio on sorghum softness and overall eating quality. These trials indicated that the grain-to-water ratio during rice-cooker cooking was a critical factor affecting sensory quality. A grain-to-water ratio of 1:4 was identified as the most suitable and was therefore selected for this study. The final cooking procedure is described below. The sorghum grains were weighed and washed with water for 2 min and repeated twice. The cleaned grains were left to drain water for 5 min. The washed and drained grains were placed in a dry and cleaned aluminum pan inside the rice cooker (Aroma rice cooker 2 quarts, Aroma Housewares, San Diego, CA, USA). Before use, the rice cooker was cleaned and verified to be in proper working condition. Appropriate food safety guidelines were followed during preparation and cooking. Grain and water were added to the rice cooker at a 1:4 ratio. The quantity of sorghum and water was also adjusted as needed based on the capacity of the rice cooker and following the instructions. The cooker was closed and the thermostat button was pressed. The sorghum was allowed to cook until the thermostat button automatically switched from the cooking to warming position. The warming light indicated that the sorghum was cooked and ready to be served. A visual representation of the nine cooked sorghum grain samples is provided in Figure 1.

### 2.2. Consumer Study—Test Design

The consumer test in this study was divided into two sessions (Table 1). Session 1 included five varieties (LUB24112, LUB24102, UC24004, UC24007 and UC24059) and Session 2 included another set of four varieties (LUB24017, UC24109, UC24001 and UC24011). Samples were randomly assigned to each batch, and the assignments were made without knowledge of the sorghum grain phenotypic information.

The test ballot included 25 questions of liking and intensity levels, which were presented on iPads programmed with Compusense software (Cloud version 19, Guelph, ON, Canada) (Table 1). These questions addressed six attributes for liking (overall, overall aroma, overall taste, overall texture, after taste, and mouthfeel), using a 9-point hedonic scale, six aroma attributes (nutty, earthy, starchy, fresh, fruity, floral, grassy, and musty), six taste and mouth sensation attributes (sweet, umami, bitter, brand, astringent, and sourness), and five texture attributes (hardens, stickiness, “residual particles left in the mouth,” roughness, and “cohesiveness to lips”), with their intensity rated using a 0–10 line scale. The line scale contained pips, in which 0 was anchored on the left end, 5 in the middle, and 10 on the right end. In addition, a demographic questionnaire consisting of six questions was included, which collected information particularly related to “preferred forms of sorghum products” and “perceived quality attributes of sorghum products” in general.

### 2.3. Consumer Study—Test Procedure

Consumer study procedures were reviewed and approved by the Texas Woman’s University Institutional Review Board (IRB), with approval IRB-FY2024-413. The participants were recruited through the TWU email listserv using flyers sent to university students, faculty, and staff. A prescreening form with a QR code was included in flyers to determine eligibility. Participation was voluntary and there were requirements for participants to be age 18 and older, not pregnant and to have no known allergy to sorghum. Eligible participants were notified via email and scheduled to participate in the test. A panel of 110 participants evaluated the sorghum grain samples in Session 1 and a separated panel of 110 participants evaluated the samples in Session 2, with around 60–70 participants attending both sessions. Participants in both batches were predominantly young adults and female with generally similar age and gender distributions.

Cooked sorghum grain samples were presented on the tray along with crackers, napkins, drinking water, individual usernames, and passwords to log into the iPad. Sorghum grain samples were cooked in the early morning before the sensory evaluation sessions began. After cooking, the samples were kept warm in a rice cooker until participants arrived. Approximately 15–20 g of each sorghum sample was then served in a 2 oz portion cup for evaluation. The samples were assigned randomly with 3-digit codes using Compusense software. The panelists were instructed to consume the sample and rate the 25 questions in the test ballot. Following evaluations of all samples in a batch, participants completed an exit survey on demographic information. The data were collected and recorded on iPads via Compusense. The completion time was designed to be around 30 min. Once fully completed, participants received petty cash as an honorarium.

### 2.4. Statistical Analysis

First, an independent-samples *t*-test was conducted to assess potential batch effects by comparing each sensory attribute between the two batches. No significant differences were observed between batches for any of the 25 attributes (*p* ≤ 0.05). Therefore, data from the two batches were pooled for subsequent analyses. Analysis of Variance (ANOVA) was performed to compare the results for each attribute across the nine sorghum grain samples using SPSS (version 25, IBM SPSS Statistics, Armonk, NY, USA). Tukey’s Honest Significant Difference (HSD) test was performed for pairwise comparisons. In addition, Pearson’s correlation analysis, partial least square (PLS) regression, and principal component analysis (PCA) were conducted using XLSTAT 2019 (Addinsoft, New York, NY, USA). Pearson’s correlation analysis was used to examine the relationships between consumer overall liking and the other 24 sensory attributes, including both hedonic and intensity ratings. PLS regression was performed with overall liking as the response variable and the remaining 24 sensory attributes as predictor variables to identify key drivers of consumer acceptance. PCA was conducted using all 25 sensory attributes to characterize and differentiate the sensory profiles and hedonic ratings of the nine sorghum grain samples. Statistical differences were considered significant with a *p*-value *≤* 0.05.

## 3. Results

### 3.1. Hedonic Rating of Cooked Sorghum Grain

A hedonic evaluation was conducted to assess consumer acceptability of the nine cooked sorghum grain samples across six sensory attributes. The liking scores of all nine samples for all six hedonic attributes ranged from 4.5 to 6.13 on a 9-point hedonic scale (Table 2), indicating overall positive acceptance. Significant differences among samples were observed for all six hedonic attributes (*p* ≤ 0.05).

Overall liking scores across the nine samples ranged from 4.63 to 5.95. LUB24102 (5.95), LUB24107 (5.89), and LUB24112 (5.88) received the highest overall liking scores, whereas UC24007 (4.63) had relatively lower consumer acceptance. Overall aroma liking scores across the nine samples ranged from 5.41 to6.13. Samples such as LUB24102 (6.13), LUB24112 (5.89), and UC24004 (5.87) received the highest score while UC24007 (5.41) received the lowest. Overall taste liking scores for all samples ranged from 5.01 to 6.06. LUB24102 (6.06) received the highest score, followed closely by LUB24112 (5.87) and LUB24107 (5.80), while UC24007 (5.01) received the lowest score. Overall texture liking scores across the nine samples ranged from 4.64 to 5.92. LUB24102 (5.92) received the highest score, followed by LUB24112 (5.68) and UC24059 (5.66), while UC24007 (4.64) and UC24011 (4.93) received the lowest score, below the middle point. Aftertaste liking scores across the nine samples ranged from 4.73 to 6.03. LUB24102 (6.03) received the highest score, followed by LUB24112 (5.74) and UC24004 (5.73), while UC24109 (4.73) received the lowest score, below the middle point. Mouthfeel liking scores across the nine samples ranged from 4.50 to 5.66. LUB24102 (5.66) received the highest score, followed by LUB24107 (5.51), while UC24007 (4.50), UC24011 (4.75), and UC24109 (4.90) had scores below 5.0. Overall aroma and taste were rated more favorably than overall texture, aftertaste, and mouthfeel across all sorghum grain samples. LUB24102, LUB24112, and LUB24107 consistently received higher liking scores across the six hedonic attributes, whereas UC24007, UC24109, and UC24011 received relatively lower scores.

Based on these results, when consuming cooked whole grain, participants preferred seeds with normal-to-hard kernels, white-to-yellow grain color, and higher starch contents. Among samples that were generally liked, the presence (LUB24107) or absence (LUB24102, LUB24112) of the waxy allele (low amylose content) did not appear to have a large impact. The presence of tannins in UC24007 was strongly disliked. Soft seeds were also disliked. Interestingly, the presence of the sugary allele in UC24109 did not improve flavor ratings, possibly because it also had relatively softer seeds and lower starch content (Table A1).

### 3.2. Sensory Attribute Intensity of Cooked Sorghum Grain

#### 3.2.1. Aroma Intensity of Cooked Sorghum Grain

The aroma profiles of the nine cooked sorghum grain samples were evaluated using eight attributes (Table 3). Overall, starchy (mean = 5.21), earthy (mean = 5.11), and nutty (mean = 5.01) aromas received the highest intensity ratings, indicating that these attributes were the dominant contributors to the aroma profiles of the samples. Fresh (mean = 4.09), grassy (mean = 3.67), and musty (mean = 3.30) aromas received moderate intensity ratings, suggesting a minor contribution to the overall aroma. In contrast, fruity and floral aroma attributes received intensity ratings below 2.5 on the 0–10 line scale, indicating minimal contribution to the overall aroma profile; therefore, they are not discussed further. Significant differences among the nine samples were observed for starchy, fruity, and musty aroma attributes (*p* ≤ 0.05).

Starchy intensity scores across all nine cooked grain samples ranged from 4.87 to 5.73. LUB24107 (5.73) showed the highest intensity followed by UC24001 (5.42) and UC24011 (5.38), while LUB24102 (4.87) and UC24007 (4.95) had the lowest. This suggests that participants detected starch more readily in softer seeds than in harder seeds, independent of the presence (LUB24107, U24011) or absence (UC24007) of the *waxy* allele (Table A1). Earthy intensity scores across both batches ranged from 4.79 to 5.43, with UC24007 (5.43), UC24109 (5.40), and UC24011 (5.38) showing the highest intensity, while four samples had an earthy intensity below 5.0, including LUB24102 (4.79), UC24004 (4.81), UC24059 (4.86), and LUB24112 (4.87). Nutty intensity scores across the nine samples ranged from 4.71 to 5.38. UC24109 (5.38) had the highest intensity, while four samples were rated below 5.0, including LUB24112 (4.71), followed by UC24004 (4.73). Overall, these three aromas were the key attributes associated with cooked sorghum grain. No clear relationships were observed between the grain color or physiochemistry profile and earthy or nutty intensity.

Fresh intensity scores across the nine samples ranged from 3.86 to 4.48, with LUB24112 (4.48) showing the highest intensity and UC24109 (3.86) the lowest. Grassy intensity scores across the nine samples ranged from 3.21 to 3.89, with UC24001 and UC24011 showing the highest intensity (3.89) and LUB24112 the lowest (3.21). Musty intensity scores across the nine samples ranged from 2.71 to 3.85, with UC24001 showing the highest intensity (3.85) and LUB24102 the lowest (2.71). Overall, fresh, grassy, and musty notes were relatively similar across different samples.

#### 3.2.2. Taste Intensity of Cooked Sorghum Grains

Six taste-related attributes were evaluated across the nine cooked sorghum grain samples (Table 3). Blandness was rated the highest among all taste attributes (mean = 6.08), followed by umami (mean = 3.45). Astringency (mean = 2.92), bitterness (mean = 2.71), and sweetness (mean = 2.78) received moderate intensity ratings. Sourness intensity ratings were minimal for all samples, indicating a negligible contribution to the overall taste profile; therefore, this attribute is not discussed further. Significant differences among the nine samples were observed for sweet, bitter, astringency, and sourness (*p* ≤ 0.05).

Bland intensity scores across the nine samples ranged from 5.69 to 6.53, with UC24004 (6.53) and UC24007 (6.48) showing the highest intensity and UC24011 (5.69) the lowest. Blandness was relatively prominent across all nine samples. Umami intensity scores across the nine samples ranged from 3.10 to 3.80, with UC24001 showing the highest intensity (3.80) and UC24007 the lowest (3.10).

Astringent intensity scores across the nine samples ranged from 2.58 to 3.57, with UC24109 showing the highest intensity (3.57) and LUB24102 and UC24004 the lowest (2.58). Bitter intensity scores across the nine samples ranged from 2.09 to 4.20, with UC24109 showing the highest intensity (4.20) and LUB24102 the lowest (2.09). Sweet intensity scores across the nine samples ranged from 2.23 to 3.26, with UC24011 showing the highest intensity (3.26) and UC24007 the lowest (2.23). Overall, these taste-related attribute intensities were relatively low.

#### 3.2.3. Texture Intensity of Cooked Sorghum Grain

Five texture-related attributes were evaluated for the nine cooked sorghum grain samples (Table 3). Residual particles received the highest intensity ratings (mean = 5.50), followed by hardness (mean = 4.40), roughness (mean = 4.20), and stickiness (mean = 3.78). “Adhesiveness to lips” was rated lowest (mean = 2.79), with moderate intensity scores. Significant differences among samples were observed for all five texture attributes across the nine samples (*p* ≤ 0.05).

Residual particles scores across the nine samples ranged from 5.10 to 6.12, with UC24007 (6.12) and UC24011 (6.04) showing the highest intensity and LUB24107 (5.10) the lowest. Hardness intensity scores across the nine samples ranged from 3.40 to 5.96, with UC24007 showing the highest intensity (5.96) and LUB24102 the lowest (3.40). Similarly, roughness intensity scores across the nine samples ranged from 3.30 to 5.55, with UC24007 showing the highest intensity (5.55) and LUB24102 the lowest (3.40). This result makes sense because UC24007 has glumes that tightly adhere to the caryopsis and cannot be completely removed during seed cleaning.

Stickiness intensity scores across the nine samples ranged from 2.91 to 5.81, with UC24011 showing the highest intensity (5.81) and UC24007 the lowest (2.91). “Adhesiveness to lips” scores across the nine samples ranged from 2.40 to 3.84, with UC24011 (3.84) showing the highest intensity, while all remaining eight samples had scores below 3.0. These results are unsurprising, as UC24011 has the *waxy* allele and the lowest amylose starch content among all samples (Table A1).

### 3.3. Sensory Attributes Driving Overall Liking of Cooked Sorghum Grain

Pearson’s correlation analysis was conducted for the nine cooked sorghum grain samples to determine the relationships between overall liking and the other 24 sensory attributes, as shown in Table 4. Overall liking demonstrated strong-to-extremely strong positive correlations with the five other hedonic ratings, including overall aroma (r = 0.807), overall taste (r = 0.969), overall texture (r = 0.922), aftertaste (r = 0.748), and mouthfeel (r = 0.964). Two sensory attribute intensities showed significant positive correlations with overall liking: fresh aroma (r = 0.544) and sweet taste (r = 0.554). In contrast, the intensity of several sensory attributes showed significant negative correlations with overall liking. The intensity of earthy (r = −0.712), grassy (r = −0.617), and musty (r = −0.597) aromas, the intensity of bitterness (r = −0.516), mouthfeel intensity attributes such as astringency (r = −0.538), and texture intensity attributes such as hardness (r = −0.911), residual particles (r = −0.931), and roughness (r = −0.962) were strongly negatively correlated with overall liking. These results indicate that breeders should not focus solely on flavor; they should also consider the physical and chemical composition of the grain, as well as threshability traits, during the development of culinary sorghums for direct consumption.

In addition to the individual correlation between attributes and overall liking, it is practical to investigate the drivers for consumers’ overall acceptance, which should correlate with multiple variables. To investigate the endogenous sensory attribute associated with overall acceptance, PLS regression was performed. Based on the PLS output, the first two components obtained from the analysis were used for the regression, as their R^2^ and Q^2^cum values (R^2^ = 0.913, Q^2^cum = 0.713, and RMSE = 0.119) indicated a good fit model.

**Figure 2:**
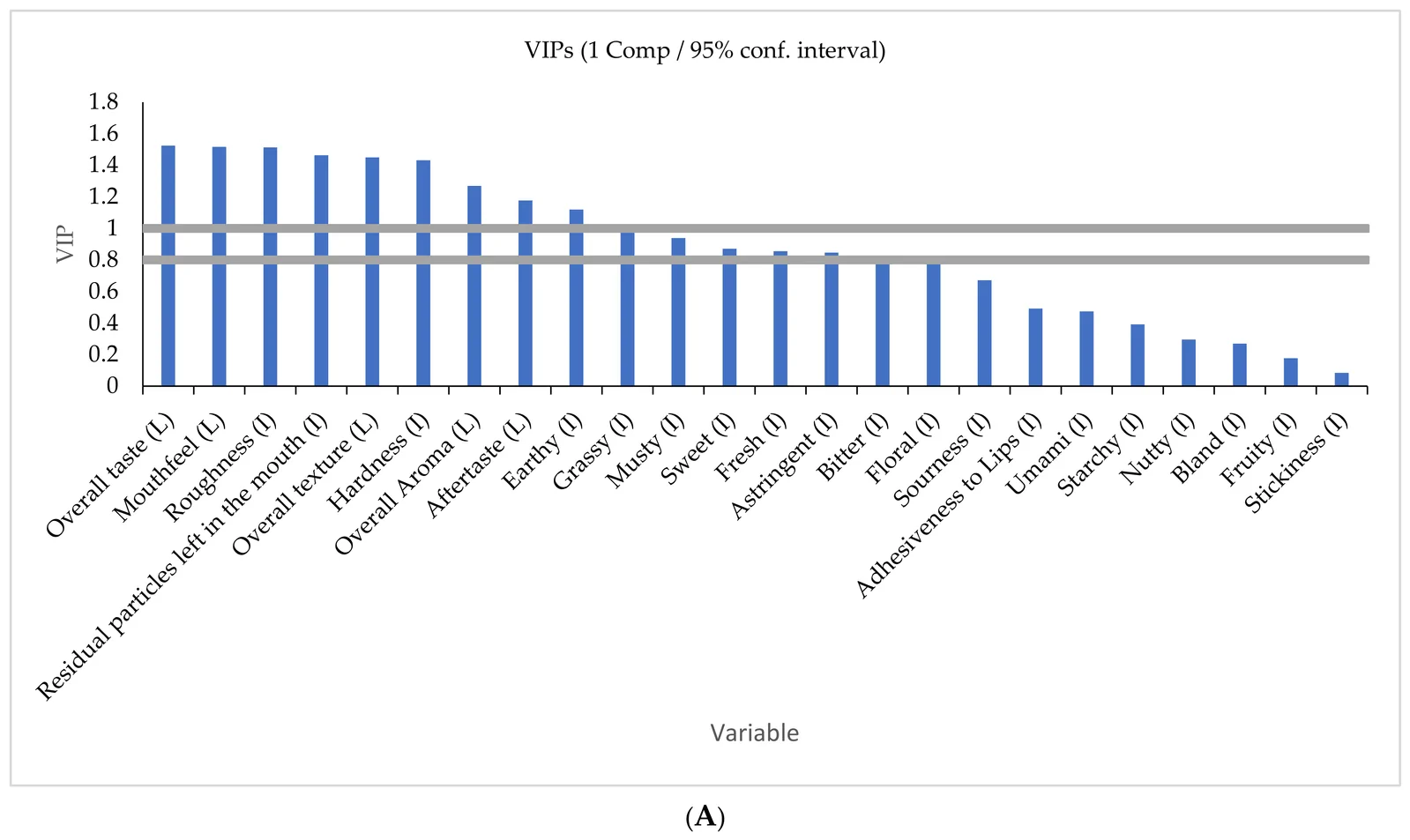

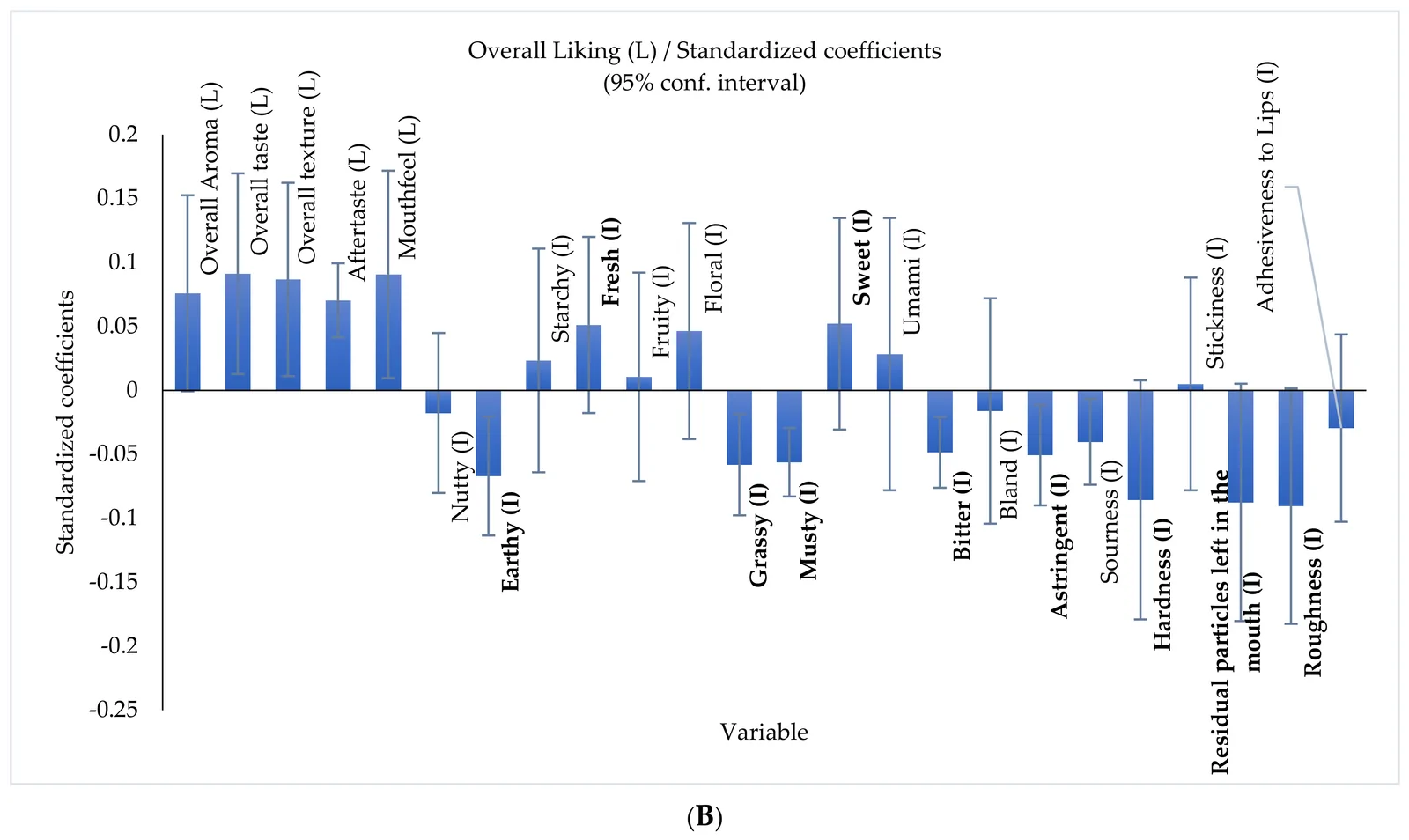
Partial least square (PLS) regression for the associations of “overall liking” and all remaining attributes’ likings and intensities for the nine cooked sorghum grain samples, including (**A**) variable importance in projection (VIP) of certain attributes and (**B**) the standardized coefficients of each attribute used to predict “overall liking”. Prediction parameters were R^2^ = 0.913, *Q*^2^cum = 0.713, and RMSE = 0.119, indicating a reasonably good fit model.

The overall acceptance of the nine cooked sorghum grain samples was highly correlated with 15 attributes (out of 24, selected with VIP > 0.8), as shown in Figure 2A. In addition to those five hedonic ratings that contributed highly and positively to overall liking, overall liking was driven by the intensities of 10 sensory attributes. Ranked from high to low VIP, these attributes were roughness, residual particles, hardness, earthy, grassy, musty, sweet, fresh, astringent, and bitter.

To weigh the contributions of each attribute to overall acceptance in the regression model, the standardized coefficients with a 95% confidence interval of PLS are displayed in Figure 2B, in which a larger coefficient indicates a more important driver. The results indicate that overall liking was most negatively influenced by aroma attributes (earthy, grassy, and musty) and mouth sensations (bitter, astringent, hardness, residual particles, and roughness). In contrast, fresh aroma and sweet taste contributed positively to overall liking.

### 3.4. Sorghum Grain Variety Difference

**Figure 3:**
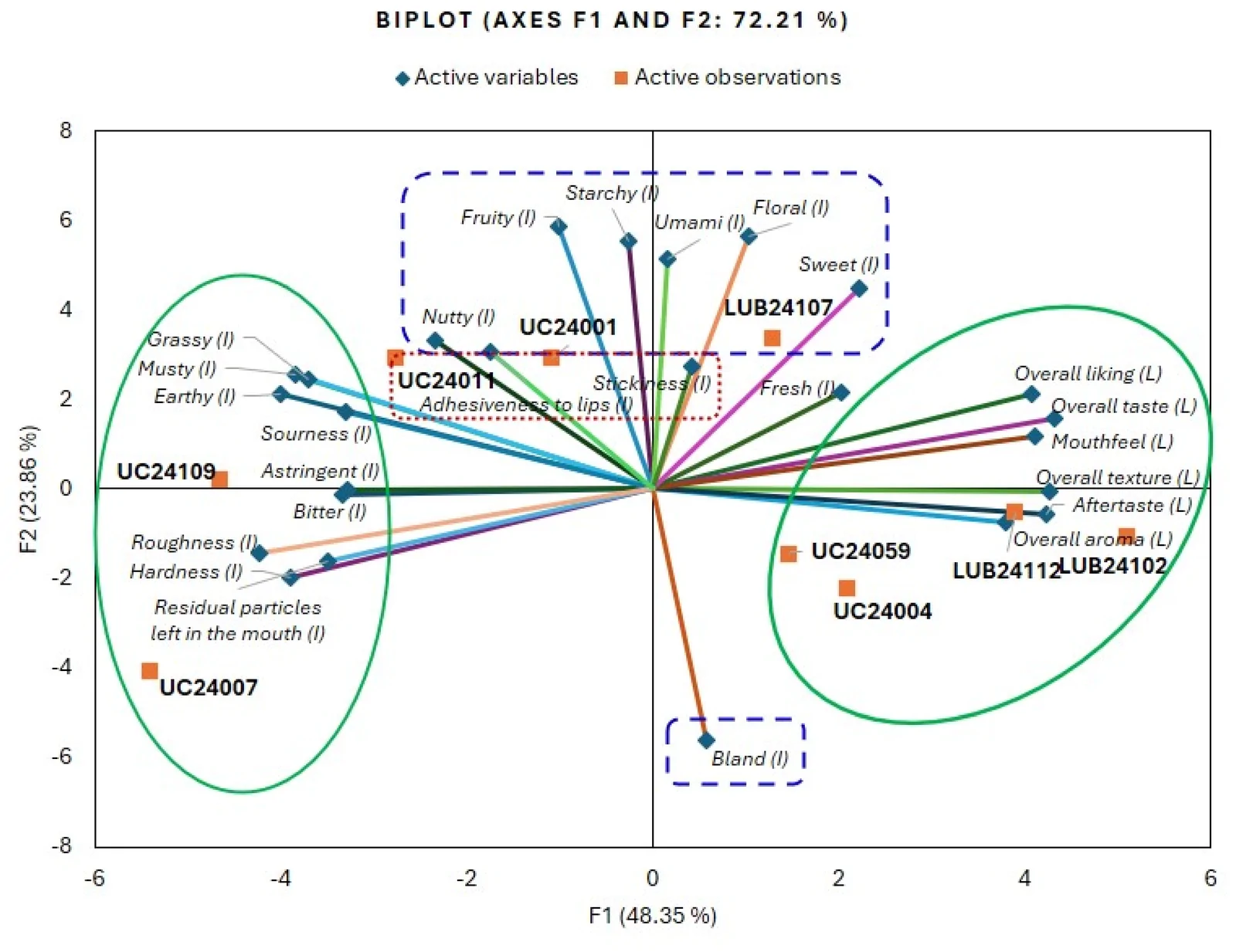
A principal component analysis (PCA) biplot of the mean scores of 25 attribute ratings for the 9 sorghum samples. L: liking; I: intensity. According to the PCA output of “Squared cosines of the variables” and “Squared cosines of the observations,” two oval circles with solid lines and three rectangle with different dash lines were created based on variables (attributes) and observations (samples) significantly separated from other samples either on the positive or negative side of PC_1_, PC_2_, and PC_3_.

To reduce the complexity of the sensory data and visualize relationships among sorghum grain samples and sensory attributes simultaneously, in this study, PCA was performed to visualize the underlying relationships between 25 attributes (loadings) and the 9 samples. The first two PCs accounted for 72.21% of the total variance, with the PC_1_ axis serving as the major component (48.35%) that differentiated samples by their attributes (Figure 3).

Four samples (LUB24102, LUB24112, UC24004, and UC24059) were separated on the positive side of PC_1_, possessing high scores in acceptance of all six attributes. These samples have bronze, white, or yellow seed, no tannins, no adhering glumes, hard kernels, and normal grain chemistry profiles. In contrast, two samples (UC24007 and UC24109) were separated on the negative side of PC_1_, which were characterized by high intensities of three aromas (grassy, musty, earthy) and six mouth sensations (sour, astringent, bitter, rough, hard, and residual particles). These samples either have tannins or adhering glumes and received low liking scores because of the high intensities of the nine attributes. In contrast, the four samples on the positive side of PC_1_ were preferred due to their low intensities of the same nine attributes.

Two samples (LUB24107 and UC24001) were separated on the positive side of PC_2_. These samples exhibited high intensity ratings for six attributes (nutty, fruity, floral, umami, sweet, and starchy) but the lowest rating for blandness. Their hedonic ratings were distinct from those of the six samples separated along PC_1_. LUB24107 has yellow grain whereas UC24001 has light-purple grain. These breeding lines may contribute novel flavor profiles with good consumer acceptance. Finally, sample UC24011 was separated along PC_3_ as it had high intensity ratings for stickiness and “adhesiveness to lips” due to the waxy allele, resulting in the lowest amylose content among all samples (Table A1). UC24011 (A.N641 x R.N642) is a high-yielding, publicly available, bronze-seeded waxy hybrid.

### 3.5. Demographic Information—Sorghum Consumption Format and Driving Factors

As the present study focused exclusively on whole sorghum grain prepared by steaming, similar to the preparation of steamed rice, the findings primarily reflect consumer perceptions and acceptance of sorghum in this specific form. However, sorghum can be consumed or incorporated into foods in a variety of other forms. To obtain a broader understanding of consumer interest in sorghum beyond the products evaluated in this study, the demographic questionnaire included queries regarding participants’ preferred forms of sorghum consumption.

**Figure 4:**
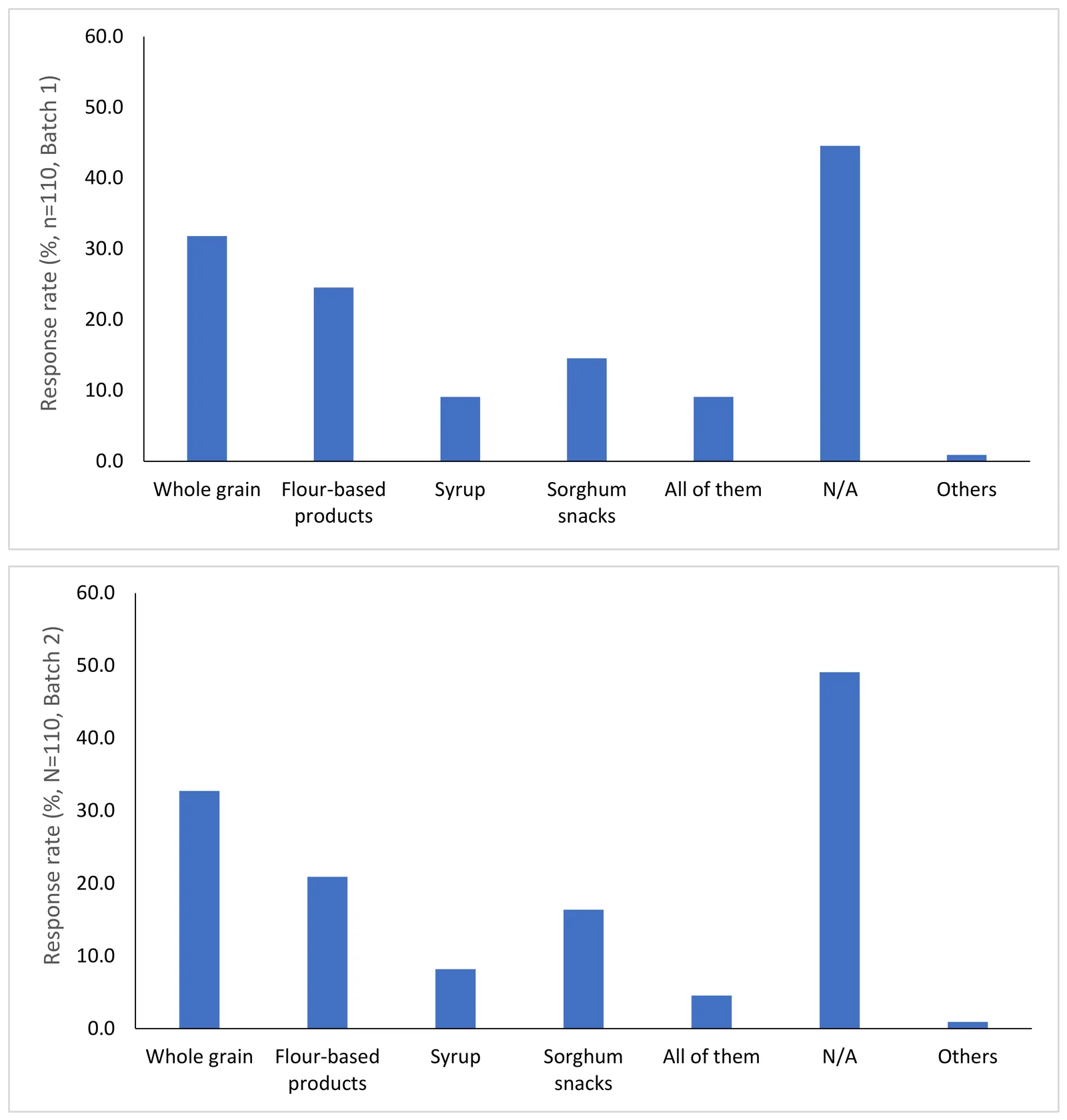
Consumer response rate (%) for the demographic question of “preferred forms of sorghum products” in general.

The results are shown in Figure 4. In Batch 1, 32% of panelists (*n* = 35) indicated a preference for sorghum in whole-grain form, 25% (*n* = 27) preferred flour-based products, while (15%, n = 16) preferred sorghum as snacks. Notably, 45% of participants (*n* = 49) reported that they did not regularly consume sorghum. Similarly, in Batch 2, 33% of panelists (*n* = 36) preferred whole-grain sorghum, followed by flour-based products (21%, *n* = 23), and snacks (16%, *n* = 18). Nearly half of the participants (49%, *n* = 54) reported that they did not regularly consume sorghum. Overall, the preference patterns were similar between the two batches, with whole-grain sorghum being the most preferred form among the specified sorghum products.

**Figure 5:**
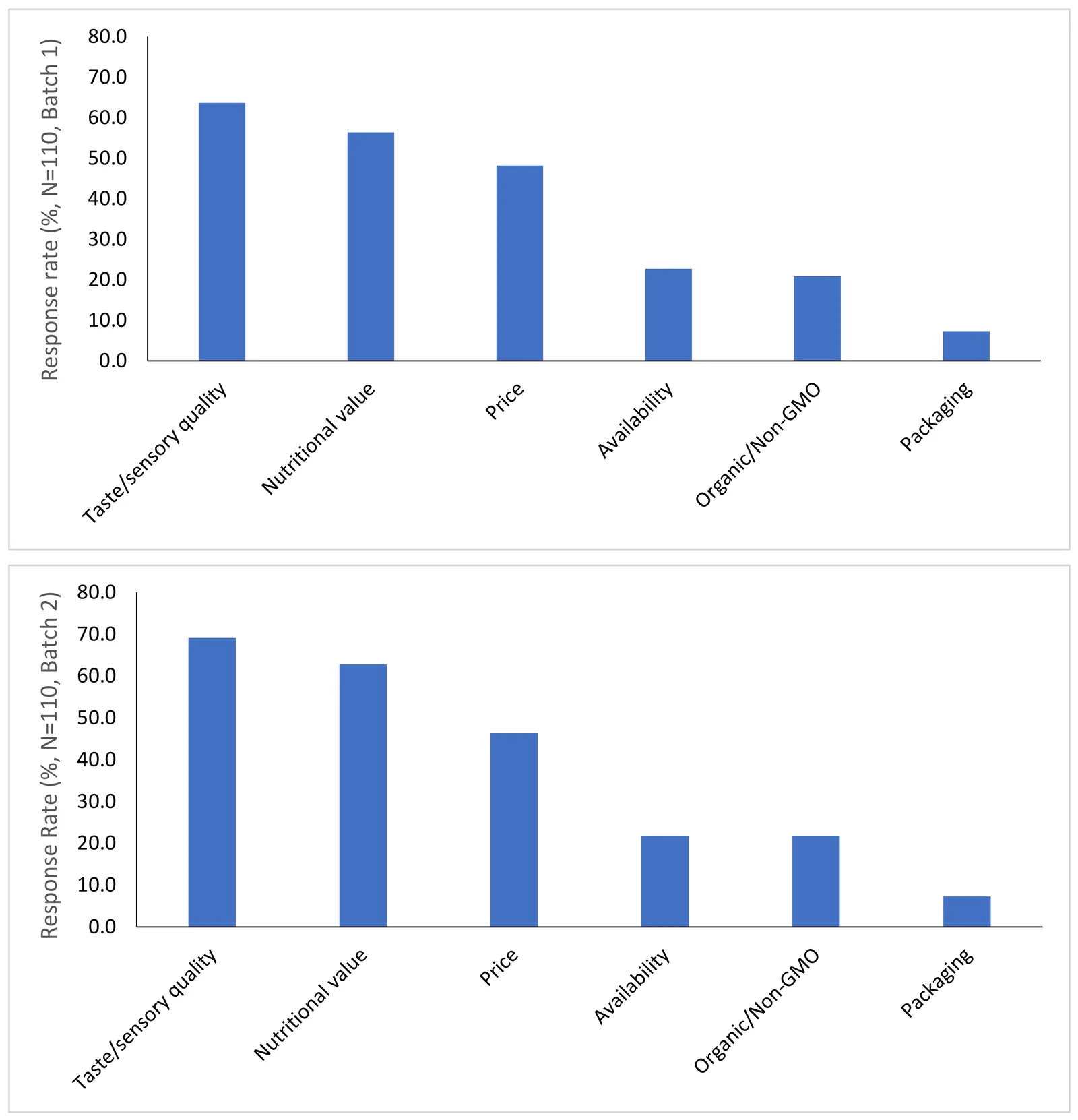
Consumer response rate (%) for the demographic question of “perceived quality attributers of sorghum products” in general.

Furthermore, data on consumer purchase intent were collected to evaluate potential factors influencing the purchase of sorghum products, as shown in Figure 5. In Batch 1, taste or sensory quality was the most frequently cited factor influencing purchase decisions, reported by 64% of participants (*n* = 70), followed by nutritional value (56%, *n* = 62) and price (48%, *n* = 53). Market availability was cited by 23% (*n* = 25), while 21% (*n* = 23) considered organic or non-GMO status important. A similar pattern was observed in Batch 2, with taste or sensory quality (69%, *n* = 76) being the most frequently cited factor, followed by nutritional value (63%, *n* = 69) and price (46%, *n* = 51). Market availability and organic or non-GMO status were each cited by 22% of participants (*n* = 24). Overall, the two batches showed similar purchase considerations, with taste or sensory quality, nutritional value, and price emerging as the primary factors influencing potential purchases of sorghum products.

## 4. Discussion

### 4.1. Consumer Acceptance and Preference of Cooked Sorghum Grain

No previous studies have evaluated the sensory characteristics or consumer acceptance of the nine samples described in this study. Therefore, direct comparison with the literature is limited. Of the six hedonic attributes evaluated, overall liking represents an integrated consumer response and is generally associated with the liking of individual sensory dimensions, including aroma, taste, texture, aftertaste, and mouthfeel. There are also important relationships among these variables themselves. For example, texture liking and mouthfeel liking are often highly correlated as both reflect consumers’ responses to the physical and oral processing characteristics of a food. Taste liking and aftertaste liking can also be strongly related because compounds responsible for taste may persist after swallowing, while aroma liking and taste liking may correlate because consumers often perceive aroma and taste together as an integrated flavor experience. In this study, the strong positive correlations among the liking of these six attributes suggest that these sensory dimensions were important contributors to consumer acceptance of cooked sorghum grains, and consumer acceptance of steamed whole sorghum was driven primarily by the integrated eating experience.

The hedonic rating findings in this study indicate that the cooked sorghum grain samples were generally acceptable to consumers. Overall aroma and taste drive acceptability, suggesting that consumers preferred steamed whole sorghum grains with a relatively mild aroma and taste profile. Samples that were less liked based on overall texture and mouthfeel are unlikely to be useful in culinary sorghum breeding due to firmer and coarser oral sensations than more commonly consumed cooked grains.

Sensory evaluations indicate that consumer acceptance of sorghum-based products depends heavily on a soft cooked texture, mild sweetness, and a clean, balanced flavor with no bitter or astringent off-notes [18,35,36]. Texture and mouthfeel are particularly critical drivers of acceptance [37,38]. Research on sorghum porridges and whole-grain sorghum foods consistently reports that samples with softer cooked textures and fewer residual particles receive higher hedonic scores, while coarse or firm grains receive lower acceptability [39,40,41]. Prior studies also note that while sorghum products often receive moderate liking on a whole-grain basis, optimized processing of softer endosperm varieties can significantly improve liking [24,42]. This suggests that commercial development of culinary sorghums may require separate breeding pools based on projected end-uses: hard kernels for direct consumption versus softer kernels for processing into flour. Our results support this view, as the softer-seeded varieties (< 70 SKCS index) performed worse when consumed as whole grains. Wheat breeding is also partitioned into soft and hard winter and spring wheat improvement programs.

Regarding overall aroma acceptance, similar findings have been reported in sorghum porridge studies [34], where aroma liking contributed to product differentiation but played a secondary role compared to texture and taste in driving overall acceptance. Prior studies show that natural sweetness enhances acceptability in whole-grain sorghum porridges and baked products [43]. Conversely, bitterness and astringency decrease consumer liking when present even at a moderate intensity [34,44]. Thus, the trends observed in the rating of six hedonic attributes for the nine cooked sorghum grain samples in this study are consistent with those reported in the literature for sorghum-based and other cereal-based products.

The variation in consumer responses among the nine cooked sorghum grain samples highlights opportunities for breeding programs to improve eating quality, particularly texture and mouthfeel, which is a key factor for increasing the consumer acceptance of cooked whole-grain sorghum. Prior sorghum sensory studies have shown that cooked sorghum grains differ widely in hardness, cohesiveness, and particle residue due to genotype-dependent differences in starch structure, endosperm hardness, and fiber content [31,45,46]. Consistently, the findings in this study indicate that consumer acceptance was strongly dependent on sorghum genotype. Several LUB24 lines (e.g., LUB24102, LUB24112, LUB24107) show consistently favorable performance across multiple sensory dimensions. In particular, LUB24102 showed broad acceptance across overall aroma, taste, texture, aftertaste, and mouthfeel, suggesting that its higher overall acceptance resulted from a favorable combination of sensory characteristics rather than a single dominant attribute. LUB24112 and LUB24107 also showed relatively favorable acceptance, further suggesting that certain breeding lines possess sensory characteristics desirable for whole-grain consumption. In particular, the low amylose content of LUB24107 suggests that it could contribute to both whole-grain consumption and processing end-uses. In contrast, the relatively lower acceptance of UC24007 and UC24109 suggests that the physical properties of the grain constrains eating quality and consumer acceptance even when aroma remains acceptable (e.g., from the sugary allele in UC24109). Interestingly, the two bronze hybrid cultivars, Richardson Seeds ‘G6’ and A.N641 x R.N642 (UC24059 and UC240110, respectively), received moderate-to-relatively low hedonic ratings across the six liking attributes compared with the seven breeding lines. This finding suggests that several lines in UNR’s culinary sorghum breeding program have achieved superior consumer acceptance to presently available varieties on the market, highlighting their commercial potential for improved sensory quality and consumer appeal.

It is well established that consumer hedonic responses are closely related to the perceived intensities of sensory attributes. The intensity of specific aroma, taste, and texture attributes can positively or negatively influence liking depending on consumer preferences and the optimal intensity of each attribute. Therefore, diagnosing sensory attribute intensities can provide insight into the sensory drivers of consumer acceptance and rejection of cooked sorghum grains.

### 4.2. Sensory Profile and Characteristic of Cooked Sorghum Grain

#### 4.2.1. Aroma Intensity

Sorghum grain has a complex sensory profile, with studies identifying more than 30 different aroma characteristics. When sorghum is cooked in water or prepared as porridge, its aroma comes from natural compounds in the grain, lipid oxidation, and compounds formed during heating, including those produced by the Maillard reaction [47,48,49]. For example, cooked sorghum descriptive terms can include cooked, floral, corn-like, green, dark green, dusty, musty, earthy, nutty, wheat-like, woody, bran, brown, buttery, cardboard, fermented, heated oil, raw, and beany [48,49]. Aroma terms such as cereal/grainy, earthy, nutty, green/grassy, and cooked grain are commonly used to describe steamed whole sorghum [49]. They describe the intrinsic grain rather than aromas introduced by processing steps such as baking and roasting. In general, nutty and earthy notes are characteristic of roasted, whole-grain sensory profiles [49,50]. Nutty aromas are commonly associated with roasted or cooked grains and may contribute to a familiar and appealing flavor profile [50].

In the present study, eight aroma attributes were selected based on our preliminary lab trials. The results showed that the aroma profile of the nine sorghum samples was primarily characterized by starchy, earthy, and nutty notes, reflecting the typical cereal- and grain-like characteristics expected from cooked whole grains. In comparison, fresh, grassy, and musty notes played secondary roles, while fruity and floral characteristics contributed little to the overall aroma. A starchy aroma is associated with cooked starch-rich foods and generally characterized as a mild, warm, cereal-like, floury, or cooked-grain aroma resulting from the heating and hydration of starches [41,43]. Starchy notes are widely documented across cereals because they arise from genotypic differences that determine how starch is chemically synthesized [31,51,52]. For earthy notes, whole grains, including sorghum, frequently present earthy or mushroom-like aromas that may be partially associated with volatile compounds like 1-octen-3-ol, generated through fatty acid oxidation in the bran fraction [45,53]. UC24109 had the highest fat content (4.27%, whole-grain, dry weight basis; Table A1) and a consistently low rating despite having a sugary allele that should have improved sweetness. This highlights the potential for some aromas to mask others and justifies the use of metabolite and/or human sensory information during breeding, rather than relying solely on a single desirable allele for flavor. Nutty aromas arise primarily from Maillard reaction products and lipid-derived volatiles during thermal processing [43,49]. Similar findings in sorghum porridge and flour indicate that compounds such as aldehydes, pyrazines, and alcohols contribute to nutty sensory attributes, which consumers often regard as indicators of desirable grain quality [45].

Fresh aromas in cereals originate from lipid oxidation derivatives, particularly hexanal and related aldehydes [41,49]. The variability observed in this study is consistent with the literature emphasizing that fresh aroma intensity depends on bran lipid composition, which differs among sorghum genotypes [45]. Grassy aromas commonly appear in cereals due to aldehydes associated with lipid oxidation [49,54]. Studies show that grassy intensity can vary by genotype depending on lipid composition and susceptibility to oxidation [41,49]. Musty notes in cereals have been linked to compounds such as geosmin or related volatiles that may arise from grain surface microbiota or bran chemistry [45]. Sorghum porridges have previously shown genotype-dependent differences in musty perception, with more intense bran flavor contributing to stronger musty notes [39,55].

The significant differences among sorghum grain samples for the intensities of several aroma attributes indicate that sorghum genotype can influence specific aroma characteristics, although the aroma profiles remained dominated by common grain-related notes. These findings suggest potential opportunities to select sorghum lines with more desirable aroma profiles for whole-grain consumption. Previous studies have shown that different sorghum varieties can have different aroma characteristics. Six Australian sorghum varieties found differences in their volatile compounds. For example, 3-octanone, which has a herbal and mushroom-like aroma, was found in Apollo, Bazley, and Liberty. The study also reported differences in compounds such as hexanal, which has a grassy aroma, and 1-octen-3-ol, which has an earthy and mushroom-like aroma [53]. Similarly, cooked waxy (sometimes called “glutinous”) and normal (“non-glutinous”) sorghum had different volatile profiles. Normal sorghum contains more types of aldehydes, while waxy sorghum contains more types of esters [49,53]. However, there was no clear relationship between liking and the presence (UC24011, UC24107) or absence of waxy alleles.

#### 4.2.2. Taste and Mouth Sensation Intensity

There are only five basic tastes, and four of these were included in this study: umami, bitter, sweet, and sour. In addition, blandness and astringency were included because they are relevant sensory characteristics of cooked sorghum and are primarily associated with chemical composition and flavor perception in the mouth rather than physical texture. Blandness reflects a low overall intensity of taste and flavor rather than a specific taste sensation, whereas astringency is considered an oral chemesthetic sensation. It is often derived from phenolic acids and tannins which are known to negatively impact the palatability of whole grain [32,56]. This was confirmed by the general disliking of UC24007. In general, astringency and bitterness negatively affect palatability when present at higher intensities, whereas sweetness may contribute positively to taste acceptance.

In this study, the taste profile of the nine cooked sorghum samples was generally mild, as reflected by the predominance of blandness and relatively low intensities of other basic tastes. The relatively high intensity of blandness indicates the mild sensory profile of the specific genotypes, aligning with sensory expectations for whole grains, where mild flavor is acceptable if textural and mouthfeel attributes are favorable [37,57]. The mild flavor profile is due to their low volatile diversity and limited presence of flavor-active compounds [45]. Blandness is a common descriptor for whole-grain sorghum porridges and cooked grains, and it reflects sorghum’s neutral base flavor, which can be considered acceptable or even desirable depending on the food application [32,41]. Umami intensity was relatively low, which is consistent with the literature indicating that sorghum is naturally low in the glutamate-related compounds responsible for the umami taste. Unlike wheat or barley products, cooked sorghum grains typically contain lower levels of free amino acids that contribute to savory or brothy sensations [31,58].

In addition, differences in astringency among samples are consistent with sorghum’s known phenolic composition, particularly the presence of tannins in UC24007, which bind to salivary proteins and cause a drying sensation [34]. Studies on African sorghum porridges report similar variability in astringency intensity across genotypes, emphasizing that tannin-rich sorghum tends to exhibit stronger astringent attributes [44]. Astringency has been frequently identified as a negative driver of consumer liking, especially when combined with elevated bitterness [41,43,49]. The bitter taste of sorghum is primarily associated with phenolic compounds, including flavonoids and, in particular, condensed tannins present in the bran layer [34]. Prior work demonstrates that sorghum varieties richer in phenolics exhibit more pronounced bitter sensations, which negatively influence consumer acceptability [44]. In contrast, variation in sweetness is most likely attributed to sorghum varieties with higher intrinsic sugar content and varied perceptions of sweetness can stem from starch digestibility and breakdown during cooking. The low amylose contents in UC24011 and LUB24107 grain were unsurprisingly associated with higher rankings for sweetness. Other studies on sorghum porridges and sorghum-based baked products have also shown that samples with higher perceived sweetness typically receive higher consumer liking [31,43]. Earlier work notes that genotypes with softer endosperms or higher soluble sugar levels may present a more pleasant sweetness profile after cooking [31]. These findings suggest that enhancing natural sweetness and minimizing bitterness and astringency through processing or breeding could improve consumer acceptance of sorghum products [32,59], as long as kernel physical characteristics are also within desirable thresholds for the anticipated end-use category (e.g., cooked whole grain vs. flour for baking).

In the current study, the consistent differences among samples in the intensity of astringency, bitterness, and sweetness indicate that these attributes are important for differentiating the taste characteristics of sorghum genotypes in UNR’s culinary sorghum breeding program. These genotype-dependent differences suggest that balancing sweetness while minimizing undesirable bitterness and astringency could be important sensory targets when selecting sorghum lines for direct whole-grain consumption. In this study, LUB24102 was perceived as the sweetest despite lacking either sugary or waxy alleles. It received the highest liking scores, suggesting the importance of multi-omics-assisted breeding approaches over Mendelian approaches to enhancing consumer preference for sorghum. In contrast, bitterness and astringency were negatively associated with liking, with higher intensities of these attributes generally corresponding to lower overall liking when consumed as a whole cooked grain, as observed for UC24007 (which has tannins and adhering glumes) and UC24109 (which combines a sugary allele with relatively soft seeds and a high fat content). Taste differences have also been reported among sorghum genotypes and grain types. For instance, Type III tannin sorghum wort showed significantly greater bitterness than other sorghum types [60], and sorghum genetics and composition, particularly tannin and phenolic content, may influence sensory quality [61].

#### 4.2.3. Texture Intensity

Texture is an important determinant of the eating quality and consumer acceptance of cooked whole grains, as it directly influences oral processing and mouthfeel. Previous sensory studies of sorghum and other cooked cereal grains have used attributes such as hardness, firmness, chewiness, roughness, graininess, cohesiveness, stickiness, adhesiveness, and residual particles to characterize texture [39,62]. These attributes reflect different aspects of grain structure, breakdown during mastication, and the sensations remaining in the mouth after swallowing. For instance, rough textures and chew resistance in whole-grain products can lead to immediate sensory rejection [63,64]. Hardness, stickiness, and residual particles are also particularly influential, with softer, less sticky, and smoother samples achieving higher acceptance [37,50,62].

In this study, five texture attributes were used to characterize the texture profiles of nine cooked sorghum samples. The results showed that the primary texture attributes were residual particles, hardness, roughness, and stickiness, reflecting the distinct oral sensations associated with consuming intact whole sorghum grains. The presence of residual particles after chewing is strongly influenced by the thickness of the pericarp (bran layer), kernel hardness, and the degree of starch swelling during cooking. Sorghum varieties with harder endosperms or higher fiber content tend to leave more coarse particles after mastication [45]. Prior sensory studies indicate that high particle residue is one of the strongest negative drivers of liking because it creates a gritty and dry mouthfeel [41,43]. Earlier reports show that some sorghum genotypes produce smoother porridges or cooked grains, while others retain coarse fragments due to their structural rigidity or incomplete hydration [31]. Hardness is also a well-established aspect of kernel structure that varies widely across sorghum genotypes. Sorghum with a harder endosperm typically resists water penetration during cooking, resulting in firmer texture and requiring more chewing effort [65]. Previous studies on cooked sorghum porridges and whole grains demonstrate that higher hardness is generally perceived as less desirable by consumers because it contributes to a tougher or drier mouthfeel [39,41,43]. Roughness is similarly explained by differences in grain composition, specifically adhesion of glumes to the caryopsis, fiber distribution, pericarp integrity, and starch–protein interactions [66,67]. Rougher textures result from incomplete swelling of grains or exposure of bran fragments, both of which are common in whole-grain sorghum [31]. Research on sorghum porridges has shown that coarse or rough textures reduce consumer liking by increasing friction in the mouth and contributing to grittiness [39,41].

Stickiness corresponds with known variations in starch gelatinization based on the amylose/amylopectin ratio, which differs among sorghum lines. Stickiness in cooked grains typically increases when starch granules swell more extensively or when amylopectin leaches during cooking [45,58]. Studies on sorghum porridges have shown that moderate stickiness contributes positively to cohesiveness and consumer preference, while extremely sticky or very loose textures may reduce acceptability depending on cultural expectations for the product [39]. The observed variability in stickiness among the present samples corresponds with underlying differences in amylose content, starch structure, and hydration properties, consistent with the literature. Adhesiveness to lips was minimal in most samples, reflecting starch behavior typical of cooked whole-grain sorghum. Adhesiveness tends to increase when starch gelatinization produces sticky surface layers, but sorghum’s relatively high amylose content and compact granule structure often limit surface stickiness compared with other cereals like rice [58]. The low-to-moderate levels of non-waxy samples in this study reflect typical textural patterns of whole cooked sorghum.

The significant differences among samples across the intensity of most texture attributes demonstrate substantial genotype-dependent variation in eating quality. For example, LUB24102 and LUB24112 had lower hardness and roughness values and were generally preferred, whereas UC24007 had high hardness and roughness and received the lowest liking scores. Given the relatively lower consumer liking for texture and mouthfeel, these differences may be particularly important in determining overall acceptance. Therefore, reducing residual particles, hardness, and roughness (due to both grain composition and threshability attributes) while maintaining an appropriate level of stickiness may represent an important target for developing culinary sorghum varieties with improved whole-grain eating quality.

It has been reported that cooking quality and softness depend strongly on genotype-specific starch–protein matrix structure and grain hydration behavior [65]. The unique starch–protein matrix of most commercially available sorghums, dominated by karifin, can create compact endosperm structures that limit water penetration during cooking [52]. Varieties that hydrate more effectively tend to gelatinize more completely, resulting in softer textures, fewer residual particles, and smoother mouthfeel attributes strongly associated with higher liking. Conversely, varieties with harder or more vitreous endosperms likely retain firmer textures and produce more perceptible particles, explaining their negative association with overall liking. These mechanisms have been reported in recent textural evaluations of sorghum kernels, where grains with denser endosperm structures exhibited higher hardness and lower sensory acceptance [25,68].

### 4.3. Sensory Drivers of Overall Liking of Cooked Sorghum Grain

Although sensory attributes differed among the sorghum varieties in this study, these attributes are likely to exhibit intrinsic relationships with one another, particularly between hedonic ratings and sensory attribute intensities. In this study, the correlation patterns observed highlight that overall liking was determined by multiple sensory dimensions and revealed the multidimensional nature of consumer acceptance of cooked sorghum grains. These findings align with previous research [23,41,62] demonstrating that texture and flavor attributes are primary determinants of liking in whole grains and cereal-based products. According to the literature [31], consumer acceptance of sorghum foods is closely tied to flavor quality and the breakdown of grain structure during cooking, which influences mouthfeel, hardness, and residual particle perception. Similarly, studies on rice and millet have shown that softness, smoothness, and reduced particle perception significantly drive consumer liking [31], supporting the pattern found in the present study.

Pearson’s correlation analysis showed the negative correlation between overall liking and the intensity of eight attributes, indicating that undesirable aromas (earthy, grassy, musty), taste/mouthfeel (bitter and astrient), and texture (hardness, residual particles, and roughness) were important sensory factors associated with reduced consumer acceptance of cooked sorghum grains. Though there is no similar sensory study for the presently evaluated culinary sorghums, prior studies on millet, quinoa, and whole-grain sorghum have similarly identified earthy or bitter notes as limiting factors in consumer acceptance [69,70]. Residual particles were particularly influential, as consumers often associate particle residues with lower processing quality or inferior product refinement [35]. In addition, harder textures have consistently been associated with lower liking, reinforcing the importance of selecting sorghum varieties with better softening characteristics upon cooking and sorghum products made from varieties with softer endosperms or a finer particle structure that produce smoother, more acceptable textures [41]. As with hardness and particle residue, roughness is a major determinant of texture-driven sensory quality in sorghum [12,71].

The PLS regression model further identified texture and mouthfeel as key drivers of acceptance, with roughness, residual particles, and hardness negatively affecting liking, indicating that varieties producing a coarse, hard, or persistent particulate sensation were generally less acceptable. The results suggest that ease of mastication and a smoother, less particulate mouthfeel are important quality targets when selecting sorghum varieties for direct consumption as cooked whole grains. In addition, aroma and taste also contributed substantially to acceptance. Earthy, grassy, and musty aromas negatively contributed to overall liking, suggesting that excessive intensities of these characteristic grain-associated notes may reduce consumer acceptance. Similarly, bitterness and astringency were negative drivers, whereas sweetness and fresh aroma contributed positively to liking. Sweetness is considered a positive sensory driver because it balances the stronger cereal notes and moderates bitterness or astringency common in whole grains [41,62].

Overall, these results suggest that consumers preferred sorghum varieties with natural sweetness and freshness, favorable texture and mouthfeel, and only mild undesirable aroma and taste notes. Thus, improving consumer acceptance of whole-grain sorghum may depend not on increasing overall flavor intensity, but rather on achieving an appropriate balance between desirable flavor characteristics and minimizing undesirable flavor and oral texture sensations.

### 4.4. Differences Among Sorghum Varieties

The differences in sorghum sample variety difference with individual sensory attributes have been thoroughly discussed in the previous sections. PCA demonstrated their value as a multivariate approach to selection that simultaneously evaluates the relationships among all 25 sensory attributes across the nine sorghum grain samples. It revealed correlations among attributes and helped to differentiate the samples based on their overall sensory profiles.

Clear varietal differences were observed in the sensory characteristics and consumer acceptance of the cooked grain samples. LUB24102, LUB24112, UC24004, and UC24059 were generally more preferred and were characterized by relatively mild aroma and taste profiles and lower intensities of undesirable texture and mouthfeel attributes. In contrast, UC24007 and UC24109 were less preferred and exhibited stronger grassy, musty, and earthy aromas, together with higher scores for sourness, bitterness, astringency, hardness, roughness, and residual particles. These contrasting profiles suggest that varietal differences in both flavor and texture can substantially influence the suitability of sorghum for consumption as steamed whole grain. Other varieties exhibited distinct sensory profiles that were not characterized simply by higher or lower overall acceptance. LUB24107 and UC24001 showed greater nutty, fruity, floral, sweet, umami, and starchy characteristics and lower blandness, indicating a more pronounced and complex flavor profile. UC24011 was distinguished primarily by greater stickiness and adhesiveness to the lips, representing a different textural characteristic from the hardness and roughness associated with UC24007 and UC24109.

Overall, these results demonstrate substantial sensory diversity among the nine sorghum varieties and highlight opportunities to select varieties with mild undesirable flavor notes, desirable flavor characteristics, and favorable texture and mouthfeel for direct whole-grain consumption.

### 4.5. Sorghum Consumption Format and Driving Factors

The demographic questionnaire regarding participants’ preferred forms of sorghum consumption provided valuable insight into how consumers may be willing to incorporate sorghum into their diets. This could guide future product development and research toward varieties of sorghum with greater consumer interest to different end-use categories. Despite its growing attention for health and sustainability benefits, regular sorghum consumption among project participants was limited. This also points to a gap between availability and consumer adoption, which can be addressed through targeted product development and consumer education.

Regarding preferred forms of sorghum consumption in general, whole grains and flour-based products were the most preferred forms across both batches. However, many participants still reported not consuming sorghum regularly, highlighting the potential to develop innovative and appealing sorghum-based products that meet modern consumer preferences [72]. This also emphasizes that consumers are more likely to accept new grains when incorporated into culturally and texturally familiar product types.

Successful market adoption of sorghum products depends on a range of interrelated consumer-driven and industry-based factors. Taste sensory quality and nutritional value were the most influential purchase drivers in both batches. Price and availability also influenced decisions, but to a lesser extent. This highlights that modern US consumers seek products that deliver both health benefits and sensory satisfactory [64,73]. Availability and labeling claims (organic, non-GMO) were less influential in the population surveyed, but still relevant, reflecting wider consumer behavioral trends towards clean labels and sustainable sourcing [74]. This emphasizes the importance of optimizing the sensory quality of sorghum products and highlights the potential of organic breeding programs to diversify the emerging culinary sorghum market.

In both batches of the study, taste or sensory quality was reported as the most important purchase driver. This reinforces the well-established principle that palatability is a non-negotiable baseline for product success, even in health or sustainability-oriented markets [75]. Nutrition ranked as the second most cited purchase motivator. Sorghum is inherently rich in fiber, polyphenols, iron, protein, and antioxidants and is naturally gluten-free, making it attractive to a growing number of consumers seeking functional foods [32]. Price was cited by nearly half of participants as a factor in their decision-making. While sorghum is often more affordable to produce than wheat or rice due to its drought resistance and low input costs, processed sorghum products may carry higher prices if not produced at scale or if labeled as specialty items. Price-sensitive consumers, such as students, families, or bulk buyers, will likely compare sorghum products with conventional grains. Hence, price competitiveness or perceived value (cost per serving) are expected to impact purchase behavior. The limited presence of sorghum products on market shelves, or unpleasant experiences with the few varieties that are commercially available, can lead to low consumer exposure and affect repeat purchases. Several consumers cited organic/non-GMO status as an influential factor. These clean-label preferences reflect growing distrust of processed foods and a shift toward natural, minimally altered ingredients [64]. All commercially available US sorghum is non-GMO and can be grown organically, adding to its appeal [64].

## 5. Conclusions

The present study provides insight into consumer perception of seven newly developed breeding lines in the culinary sorghum breeding program at UNR, alongside two hybrid cultivars, when consumed as cooked whole grain. This end-use category has received relatively limited attention compared to processed sorghum products. The findings demonstrate that the breeding sorghum lines have distinct sensory characteristics that influence their suitability for direct human consumption. The results highlight the need to consider multivariate sensory quality during the breeding process when varieties are intended for direct human consumption, as shown in PCA. The lines with superior eating characteristics included LUB24102, LUB24112, and LUB24107. Additional efforts are underway to map metabolite profiles associated with multivariate sensory data to specific genes to guide ongoing breeding. Identifying beneficial alleles associated with desirable chemical compounds and consumer-perceived sensory attributes should be a top priority for culinary sorghum breeding programs.

Potential limitations of this study include the use of a single cooking condition (a fixed 1:4 grain-to-water ratio), which may not represent the optimal preparation method for all genotypes and may disadvantage genotypes with harder kernels. In addition, because untrained consumers rated attribute intensities using a line scale in this study, these responses should be interpreted as consumer-perceived sensory intensities rather than conventional descriptive analysis measurements. Future studies using trained panels are needed to confirm and more precisely quantify these sensory differences.

## Figures and Tables

**Table 1:** Cooked sorghum grain consumer test design.

Session	Sample	Liking and Scale	Aroma Intensity and Scale	Taste Intensity and Scale	Texture Intensity and Scale
Session 1 Participants (*N* = 110)	LUB24112 LUB24102 UC24004 UC24007 UC24059	6 hedonic questions:Overall Overall aroma Overall taste Overall texture Aftertaste Mouthfeel	8 aroma intensity questions: Nutty Earthy Starchy Fresh Fruity Floral Grassy Musty	6 taste intensity questions: Sweet Umami Bitter Bland Astringent Sour	5 texture intensity questions: Hardness Stickiness Residual particles left in the mouth Roughness Adhesiveness to lips
Session 2 Participants (*N* = 110)	LUB24017 UC24109 UC24001 UC24011	9-point hedonic scale: 1 = dislike extremely 2 = dislike very much 3 = dislike moderately 4 = dislike slightly 5 = either like or dislike 6 = like slightly 7 = like moderately 8 = like very much 9 = like extremely	0–10 line scale: *Pips* 0 = none 5 = moderate 10 = extremely strong	0–10 line scale: *Pips* 0 = none 5 = moderate 10 = extremely strong	0–10 line scale: *Pips* 0 = none 5 = moderate 10 = extremely strong

**Table 2:** Hedonic rankings of six attributes among nine cooked sorghum grain samples (batch 1, *n* = 110; batch 2, *n* = 110).

	Batch 1					Batch 2				
*Hedonic Attributes*	LUB24112	LUB24102	UC24004	UC24007	UC24059	LUB24107	UC24109	UC24001	UC24011	Sig.
Overall liking	5.88 bc	5.95 c	5.66 bc	4.63 a	5.64 bc	5.89 bc	5.25 ab	5.61 bc	5.18 ab	***
Overall aroma liking	5.89 ab	6.13 b	5.87 ab	5.41 a	5.78 ab	5.72 ab	5.76 ab	5.63 ab	5.52 ab	*
Overall taste liking	5.87 bc	6.06 c	5.67 abc	5.01 a	5.67 abc	5.80 bc	5.27 ab	5.65 abc	5.39 ab	***
Overall texture liking	5.68 cd	5.92 d	5.61 bcd	4.64 a	5.66 bcd	5.59 bcd	5.10 abc	5.12 abc	4.93 ab	***
Aftertaste liking	5.74 bc	6.03 c	5.73 bc	5.08 ab	5.64 bc	5.49 bc	4.73 a	5.41 abc	5.30 ab	***
Mouthfeel liking	5.35 bcd	5.66 d	5.25 bcd	4.50 a	5.38 bcd	5.51 cd	4.90 abc	5.15 abcd	4.75 ab	***

Note: Different letters in a row indicate significant differences according to one-way ANOVA with Tukey’s HSD test (α ≤ 0.1). *: *p* ≤ 0.1; ***: *p* ≤ 0.01.

**Table 3:** Intensity ratings of eight aroma, six taste, and five texture attributes for nine cooked sorghum grain samples (batch 1, *n* = 110; batch 2, *n* = 110).

	Batch 1					Batch 2				
	LUB24112	LUB24102	UC24004	UC24007	UC24059	LUB24107	UC24109	UC24001	UC24011	Sig.
Aroma										
Nutty	4.71 a	4.88 a	4.73 a	4.88 a	5.16 a	5.15 a	5.38 a	5.12 a	5.12 a	
Earthy	4.87 a	4.79 a	4.81 a	5.43 a	4.86 a	5.29 a	5.40 a	5.20 a	5.38 a	
Starchy	5.29 a	4.87 a	5.04 a	4.95 a	5.03 a	5.73 ab	5.17 a	5.42 a	5.38 a	*
Fresh	4.48 a	3.94 a	3.99 a	3.97 a	4.04 a	4.46 a	3.86 a	4.18 a	3.89 a	
Fruity	1.83 ab	1.82 ab	1.83 ab	1.66 a	1.75 ab	2.27 ab	2.09 ab	2.60 b	2.34 ab	**
Floral	2.49 a	2.37 a	2.34 a	2.18 a	2.20 a	2.65 a	2.33 a	2.62 a	2.49 a	
Grassy	3.21 a	3.39 a	3.50 a	3.86 a	3.64 a	3.81 a	3.83 a	3.89 a	3.89 a	
Musty	2.82 a	2.71 a	3.05 ab	3.58 ab	3.10 ab	3.42 ab	3.69 ab	3.85 b	3.51 ab	**
Taste/mouthfeel										
Sweet	2.92 ab	3.15 b	2.57 ab	2.23 a	2.53 ab	3.01 ab	2.38 ab	2.95 ab	3.26 b	**
Umami	3.28 a	3.29 a	3.50 a	3.10 a	3.47 a	3.58 a	3.34 a	3.80 a	3.67 a	
Bitter	2.23 a	2.09 a	2.74 a	3.00 a	2.30 a	2.59 a	4.20 b	2.78 a	2.50 a	***
Bland	6.19 a	6.00 a	6.53 a	6.48 a	6.33 a	5.78 a	5.83 a	5.93 a	5.69 a	
Astringent	2.78 ab	2.58 a	2.58 a	3.19 ab	2.90 ab	3.08 ab	3.57 b	2.74 ab	2.83 ab	*
Sourness	1.25 a	1.36 a	1.34 a	1.62 ab	1.53 a	1.62 ab	2.35 b	1.78 ab	1.62 ab	***
Texture										
Hardness	3.69 ab	3.40 a	4.60 bc	5.96 d	3.97 abc	3.71 ab	4.79 c	4.86 c	4.59 bc	***
Stickiness	3.99 b	4.02 b	3.35 ab	2.91 a	3.80 ab	3.48 ab	3.38 ab	3.25 ab	5.81 c	***
Residual particles left in the mouth	5.28 ab	5.28 ab	5.33 ab	6.12 b	5.41 ab	5.10 a	5.59 ab	5.36 ab	6.04 b	**
Roughness	3.71 ab	3.30 a	4.04 ab	5.55 c	3.81 ab	3.75 ab	4.67 bc	4.41 b	4.56 bc	***
Adhesiveness to lips	2.87 ab	2.40 a	2.52 a	2.55 a	2.70 a	2.56 a	2.94 ab	2.73 a	3.84 b	***

Note: Different letters in a row indicate significant differences according to one-way ANOVA with Tukey’s HSD test (α ≤ 0.1). *: *p* ≤ 0.1; **: *p* ≤ 0.05; ***: *p* ≤ 0.01.

**Table 4:** Pearson correlation coefficients between “overall liking” and all remaining 24 sensory attributes.

	Pearson Correlation (*r*)
Hedonic	
Overall aroma liking	**0.807**
Overall taste liking	**0.969**
Overall texture liking	**0.922**
Aftertaste liking	**0.748**
Mouthfeel liking	**0.964**
Aroma	
Nutty intensity	−0.188
Earthy intensity	**−0.712**
Starchy intensity	0.249
Fresh intensity	**0.544**
Fruity intensity	0.113
Floral intensity	0.493
Grassy intensity	**−0.617**
Musty intensity	**−0.597**
Taste/mouthfeel	
Sweet intensity	**0.554**
Umami intensity	0.301
Bitter intensity	**−0.516**
Bland intensity	−0.171
Astringent intensity	**−0.538**
Sour intensity	−0.427
Texture	
Hardness intensity	**−0.911**
Stickiness intensity	0.053
Residual particles left in the mouth intensity	**−0.931**
Roughness intensity	**−0.962**
Adhesiveness to lips intensity	−0.313

Note: Correlation is significant at *p* ≤ 0.05 level. Bold numbers indicate statical significance.

## Data Availability

Data will be made available on request. The sorghum breeding lines described in this study, including the publicly available waxy sorghum hybrid (A.N641 x R.N642), are available through licensing or optioning agreements with UNR’s Technology Transfer Office. Contact Melinda Yerka for details at myerka@unr.edu.

## Appendix A

The basic composition of the nine raw sorghum grains was quantified, including moisture, protein, lysine, starch, amylose, and fat contents. UC24 samples were hand-harvested, and moisture content was determined in the laboratory via NIR analysis. LUB24 samples were combine-harvested, and moisture content was measured by the combine at harvest. Protein, lysine, starch, amylose and fat contents are given on a percent whole-grain dry-weight basis, as determined by well-calibrated NIR equations at the USDA-ARS Grain Structure & Quality Research Lab in Manhattan, KS. NIR equations were updated annually with wet chemistry. The SKCS (kernel hardness) index was based on 100 individual seeds, where higher numbers denote harder seeds. For these analyses, SKCS < 70.00 was considered normal kernel hardness and 50.00 < SKCS < 70.00 was considered medium kernel hardness. Similarly, amylose content >16.5% was considered normal, 10.0% < amylose < 16.5% was considered moderate, and < 10.0% was considered low. Protein content > 12.0% was considered high.

**Table A1:** Basic composition and physicochemical properties of nine raw sorghum grains.

Sample	LUB24112	LUB24102	UC24004	UC24007	UC24059	LUB24107	UC24109	UC24001	UC24011
Type	l	l	l	l	h	l	l	l	h
Grain phenotype	N	N	N	HP, MA	N	MA, MKH	HP, SU, MKH	HP	HP, LA, MKH
Moisture (%)	11.1	11.1	9.33	9.56	9.24	11.1	9.37	9.32	9.71
Protein (%)	9.34	10.66	11.37	14.33	10.6	10.28	13.48	12.64	13.43
Lysine (%)	0.25	0.25	0.27	0.32	0.26	0.27	0.34	0.29	0.32
Starch (%)	75.08	72.97	72.25	68.03	72.54	72	64.95	70.07	68.11
Amylose (%)	17.85	16.97	18.56	16.78	18.66	12.07	16.56	17.89	7.01
Fat (%)	3.52	2.72	2.96	2.94	3.2	3.44	4.27	2.61	3.28
SKCS	78.37	90.61	85.48	81.46	85.81	66.84	64.88	71.02	68.34
Seed diameter (mm)	2.2	2.32	2.45	2.5	2.34	2.59	2.93	2.71	2.71
Seed mass (mg)	18.86	20.62	25.09	27.93	24.05	26.51	35.53	32.57	34.57

Note: HP, high protein; N, normal; MA, moderate amylose; LA, low amylose; MKH, medium kernel hardness; SU, sugary allele; I, inbred line; h, hybrid cultivar.

## References

[1] Bakari H., Djomdi, Ruben Z.F., Roger D.D., Cedric D., Guillaume P., Pascal D., Philippe M., Gwendoline C. (2023). Sorghum (*Sorghum bicolor* L. moench) and its main parts (by-products) as promising sustainable sources of value-added ingredients. Waste Biomass Valorization.

[2] Hu W., Zhou L., Chen J. (2022). Conversion sweet sorghum biomass to produce value-added products. Biotechnol. Biofuels Bioprod..

[3] Yerka M.K., Liu Z., Bean S., Nigam D., Hayes C., Druetto D., Krishnamoorthy G., Meiwes S., Cucit G., Patil G.B. (2026). An updated molecular toolkit for genomics-assisted breeding of waxy sorghum [*Sorghum bicolor* (L.) moench]. J. Appl. Genet..

[4] Tasie M.M., Gebreyes B.G. (2020). Characterization of nutritional, antinutritional, and mineral contents of thirty-five sorghum varieties grown in Ethiopia. Int. J. Food Sci..

[5] Srivastava A.K., Riaz A., Jiang J., Li X., Uzair M., Mishra P., Zeb A., Zhang J., Singh R.P., Luo L. (2025). Advancing climate-resilient sorghum: The synergistic role of plant biotechnology and microbial interactions. Rice.

[6] Mundia C.W., Secchi S., Akamani K., Wang G. (2019). A regional comparison of factors affecting global sorghum production: The case of North America, Asia and Africa’s Sahel. Sustainability.

[7] Hossain M.S., Islam M.N., Rahman M.M., Mostofa M.G., Khan M.A.R. (2022). Sorghum: A prospective crop for climatic vulnerability, food and nutritional security. J. Agric. Food Res..

[8] Lk M., Malam K.V. (2020). Sweet sorghum: A smart, multipurpose crop. Agric. Environ..

[9] Whitfield M.B., Chinn M.S., Veal M.W. (2012). Processing of materials derived from sweet sorghum for biobased products. Ind. Crops Prod..

[10] Tanwar R., Panghal A., Chaudhary G., Kumari A., Chhikara N. (2023). Nutritional, phytochemical and functional potential of sorghum: A review. Food Chem. Adv..

[11] Ni C., Zhang S., Zhang G., Cheng J., Zheng H. (2019). Evaluation of edible quality of sorghum based on principal component analysis. J. Chem..

[12] Adzqia F., Suwonsichon S., Thongngam M. (2023). Effects of white sorghum flour levels on physicochemical and sensory characteristics of gluten-free bread. Foods.

[13] Anglani C. (1998). Sorghum for human food—A review. Plant Foods Hum. Nutr..

[14] Chavan U.D., Dalvi U.S., Pawar G.H., Shinde M.S. (2013). Selection of genotype and development of technology for sorghum hurda production. Int. Food Res. J..

[15] Pontieri P., Del Giudice L. (2016). Sorghum: A novel and healthy food. Encyclopedia of Food and Health.

[16] Miftahurrahmi, Antarlina S.S., Ginting E., Khamidah A., Purwani E.Y., Sihono, Ismail N., Yustina I., Ambarsari I., Fauziah L. (2026). Sorghum phenolic compounds: Chemistry, health benefits, processing effects, and applications in functional foods. J. Agric. Food Res..

[17] Palavecino P.M., Curti M.I., Bustos M.C., Penci M.C., Ribotta P.D. (2020). Sorghum pasta and noodles: Technological and nutritional aspects. Plant Foods Hum. Nutr..

[18] Fernando W.M.A.D.B., Abdulraheem R.A., Kariyawasam K.P., Samarasinghe A.S., Barnes H., Robertson M., Jayatunga D.P.W., De Silva B.G.D.N.K., Jayasena V. (2025). Sorghum and Sorghum-Based products: Nutritional composition, prebiotic potential and health benefits in gut microbiota interactions. Int. J. Food Sci..

[19] Seydoşoğlu S., Turan N., Kökten K., Özdemir S. (2024). Feed quality of sweet sorghum grains. Selcuk J. Agric. Food Sci..

[20] Palacios C.E., Nagai A., Torres P., Rodrigues J.A., Salatino A. (2021). Contents of tannins of cultivars of sorghum cultivated in Brazil, as determined by four quantification methods. Food Chem..

[21] Salazar-López N.J., González-Aguilar G., Rouzaud-Sández O., Robles-Sánchez M. (2018). Technologies applied to sorghum (*Sorghum bicolor* L. moench): Changes in phenolic compounds and antioxidant capacity. Food Sci. Technol..

[22] Espitia-Hernández P., Chávez González M.L., Ascacio-Valdés J.A., Medina M.D.D., Flores-Naveda A., Silva T., Ruelas-Chacón, Sepúlveda L., Espitia P. (2022). Sorghum (*Sorghum bicolor* L. moench): Chemical composition and its health benefits. New Insights in Food Research and Engineering: The Trend for World Sustainable Food Production.

[23] De Leon A., Burnett D.J., Rust B.M., Casperson S.L., Horn W.F., Keim N.L. (2020). Liking and acceptability of whole grains increases with a 6-week exposure but preferences for foods varying in taste and fat content are not altered: A randomized controlled trial. Curr. Dev. Nutr..

[24] Niba L.L., Bokanga M.M., Jackson F.L., Schlimme D.S., Li B.W. (2002). Physicochemical properties and starch granular characteristics of flour from various *Manihot esculenta* (cassava) genotypes. J. Food Sci..

[25] Zheng H., Dang Y., Sui N. (2023). Sorghum: A multipurpose crop. J. Agric. Food Chem..

[26] Bukonja S., Tomić J., Pestorić M., Maravić N., Despotović S., Tomičić Z., Kiprovski B., Pantelić N.Đ. (2025). Exploring sorghum flour as a sustainable ingredient in gluten-free cookie production. Foods.

[27] Wagaw K. (2019). Review on mechanisms of drought tolerance in sorghum (*Sorghum bicolor* (L.) moench) basis and breeding methods. Acad. Res. J. Agric. Sci. Res..

[28] Mwamahonje A., Mdindikasi Z., Mchau D., Mwenda E., Sanga D., Garcia-Oliveira A.L., Ojiewo C.O. (2024). Advances in sorghum improvement for climate resilience in the global arid and semi-arid tropics: A review. Agronomy.

[29] Hao H., Li Z., Leng C., Lu C., Luo H., Liu Y., Wu X., Liu Z., Shang L., Jing H.-C. (2021). Sorghum breeding in the genomic era: Opportunities and challenges. Theor. Appl. Genet..

[30] Motlhaodi T., Bryngelsson T., Chite S., Fatih M., Ortiz R., Geleta M. (2018). Nutritional variation in sorghum [*Sorghum bicolor* (L.) moench] accessions from southern Africa revealed by protein and mineral composition. J. Cereal Sci..

[31] Taylor J., Bean S.R., Duodu K.G. (2025). Sorghum and Millets: Chemistry, Technology, and Nutritional Attributes.

[32] Awika J.M. (2017). Sorghum: Its unique nutritional and health-promoting attributes. Gluten-Free Ancient Grains.

[33] Wang Z., Wang C., Zhang S., Ren H., Ren C. (2026). Moderate processing of rice: Processing technology, quality evaluation, and reflections precision. Foods.

[34] Awika J.M., Rooney L.W. (2004). Sorghum phytochemicals and their potential impact on human health. Phytochemistry.

[35] de Morais Cardoso L., Pinheiro S.S., Martino H.S.D., Pinheiro-Sant’Ana H.M. (2017). Sorghum (*Sorghum bicolor* L.): Nutrients, bioactive compounds, and potential impact on human health. Crit. Rev. Food Sci. Nutr..

[36] Park O.-H., Sosa-Holwerda A., Niraula S.R., Maki K., Thompson L., Moustaid-Moussa N. (2025). Exploring college students’ acceptance of and behavioral intentions toward different sorghum-based foods. Foods.

[37] Chiş M.S., Păucean A., Man S.M., Mureşan V., Socaci S.A., Pop A., Stan L., Rusu B., Muste S. (2020). Textural and sensory features changes of gluten free muffins based on rice sourdough fermented with *Lactobacillus spicheri* DSM 15429. Foods.

[38] Toth M., Vatai G., Koris A. (2020). Consumers’ acceptance, satisfaction in consuming gluten-free bread: A market survey approach. Int. J. Celiac Dis..

[39] Gobara Hamid M., Böhme C., Mustafa K.A., Idris Y.M.A., Hamad M., Muneer F., Rahmatov M., Elsafy M., Abdelhalim T. (2025). Sensory profiling of traditional sorghum porridge (*Aceda*): Advancing nutritious and culturally accepted biofortified products. Front. Sustain. Food Syst..

[40] de Oliveira L., de Oliveira G.T., de Alencar E.R., Queiroz V.A.V., de Alencar Figueiredo L.F. (2022). Physical, chemical, and antioxidant analysis of sorghum grain and flour from five hybrids to determine the drivers of liking of gluten-free sorghum breads. LWT.

[41] Bhasin T. (2020). Sensory characteristics of wholegrain. Adv. Obes. Weight Manag..

[42] Rooney L.W., Awika J.M. (2005). Overview of products and health benefits of specialty Sorghums. Cereal Foods World.

[43] Spio-Kwofie A., Agambire M.A. (2023). Development of sorghum-based shortbread biscuits. J. Exp. Food Chem..

[44] Dlamini N.R., Taylor J.R., Rooney L.W. (2007). The effect of sorghum type and processing on the antioxidant properties of African sorghum-based foods. Food Chem..

[45] Taylor J.R., Belton P.S., Beta T., Duodu K.G. (2014). Increasing the utilisation of sorghum, millets and pseudocereals: Developments in the science of their phenolic phytochemicals, biofortification and protein functionality. J. Cereal Sci..

[46] Taylor J.R., Dewar J. (2001). Developments in sorghum food technologies. Adv. Food Nutr. Res..

[47] Suwonsichon S. (2019). The importance of sensory lexicons for research and development of food products. Foods.

[48] Tran T., Chambers E. (2020). Development of a model system for tasting grain varieties. Foods.

[49] Chen S., Wang L., Ni D., Lin L., Wang H., Xu Y. (2021). Characterization of aroma compounds in cooked sorghum using comprehensive two-dimensional gas chromatography-time-of-flight mass spectrometry and gas chromatography-olfactometry-mass spectrometry. Molecules.

[50] Taylor J.R.N., Schober T.J., Bean S.R. (2006). Novel food and non-food uses for sorghum and millets. J. Cereal Sci..

[51] Khatefov E.B., Goldstein V.G., Krivandin A.V., Wasserman L.A. (2023). Main characteristics of processed grain starch products and physicochemical features of the starches from maize (*Zea mays* L.) with different genotypes. Polymers.

[52] Beta T., Corke H., Rooney L.W., Taylor J.R.N. (2001). Starch properties as affected by sorghum grain chemistry. J. Sci. Food Agric..

[53] Fan X., Jiao X., Liu J., Jia M., Blanchard C., Zhou Z. (2021). Characterizing the volatile compounds of different sorghum cultivars by both GC-MS and HS-GC-IMS. Food Res. Int..

[54] Qi S., Ren H., Yang H., Zhang L., Zhang M. (2026). Comparative study of key aroma components in rice of different aroma types using flavor metabolomics. Foods.

[55] Adebowale O.J., Taylor J.R.N., de Kock H.L. (2020). Stabilization of wholegrain sorghum flour and consequent potential improvement of food product sensory quality by microwave treatment of the kernels. LWT.

[56] Călinoiu L.F., Vodnar D.C. (2018). Whole grains and phenolic acids: A review on bioactivity, functionality, health benefits and bioavailability. Nutrients.

[57] Xhakollari V., Canavari M., Osman M. (2019). Factors affecting consumers’ adherence to gluten-free diet, a systematic review. Trends Food Sci. Technol..

[58] Smith C.W., Frederiksen R.A. (2000). Sorghum: Origin, History, Technology, and Production.

[59] Morey C., Kubota J., Anand S., Feng X. (2025). Whole grains and ancient wheats: Knowledge, attitudes, consumption behavior and sensory liking of breads among participants in the United States. Food Humanit..

[60] Adetunji A.I., Khoza S., de Kock H.L., Taylor J.R.N. (2013). Influence of sorghum grain type on wort physico-chemical and sensory quality in a whole-grain and commercial enzyme mashing process. J. Inst. Brew..

[61] Bernardo C.O., Ascheri J.L.R., Carvalho C.W.P., Chávez D.W.H., Martins I.B.A., Deliza R., Freitas D.d.G.C., Queiroz V.A.V. (2019). Impact of extruded sorghum genotypes on the rehydration and sensory properties of soluble beverages and the Brazilian consumers’ perception of sorghum and cereal beverage using word association. J. Cereal Sci..

[62] Heiniö R., Noort M., Katina K., Alam S., Sozer N., de Kock H., Hersleth M., Poutanen K. (2016). Sensory characteristics of wholegrain and bran-rich cereal foods—A review. Trends Food Sci. Technol..

[63] Kumar H., Dhalaria R., Guleria S., Sharma R., Cimler R., Dhanjal D.S., Chopra C., Kumar V., Manickam S., Siddiqui S.A. (2025). Advances in the concept of functional foods and feeds: Applications of cinnamon and turmeric as functional enrichment ingredients. Crit. Rev. Food Sci. Nutr..

[64] Aschemann-Witzel J., Varela P., Peschel A.O. (2019). Consumers’ categorization of food ingredients: Do consumers perceive them as ‘clean label’ producers expect? An exploration with projective mapping. Food Qual. Prefer..

[65] Belton P.S., Taylor J.R. (2004). Sorghum and millets: Protein sources for Africa. Trends Food Sci. Technol..

[66] Chen X., Yu J., Zhu Y., Liu G., Li G., Xu F., Hu Q., Cao J., Zhang H., Wei H. (2026). Interrelationships between appearance quality and starch and protein structures in superior and inferior grains of soft and non-soft rice. Rice.

[67] Fujimoto K., Minami N., Goto T., Ishida Y., Watanabe M., Nagao K., Ichikawa T. (2016). Hardness, cohesiveness, and adhesiveness of oral moisturizers and denture adhesives: Selection criteria for denture wearers. Dent. J..

[68] Tripathi M.K., Jadam R.S., Kumar A. (2021). Quality management system in millet and sorghum. Millets and Millet Technology.

[69] Haro-Vicente J.F., Bernal-Cava M.J., Lopez-Fernandez A., Ros-Berruezo G., Bodenstab S., Sanchez-Siles L.M. (2017). Sensory acceptability of infant cereals with whole grain in infants and young children. Nutrients.

[70] Binou P., Yanni A.E., Karathanos V.T. (2022). Physical properties, sensory acceptance, postprandial glycemic response, and satiety of cereal based foods enriched with legume flours: A review. Crit. Rev. Food Sci. Nutr..

[71] Adeyeye S.A.O. (2016). Assessment of quality and sensory properties of sorghum–wheat flour cookies. Cogent Food Agric..

[72] Jones J.M., Engleson J. (2010). Whole grains: Benefits and challenges. Annu. Rev. Food Sci. Technol..

[73] Ares G., Gámbaro A. (2007). Influence of gender, age and motives underlying food choice on perceived healthiness and willingness to try functional foods. Appetite.

[74] Grunert K.G. (2023). Consumer Trends and New Product Opportunities in the Food Sector.

[75] Prescott J. (1999). Flavour as a psychological construct: Implications for perceiving and measuring the sensory qualities of foods. Food Qual. Prefer..

